# A Set of Artificial Pollination Technical Measures: Improved Seed Yields and Active Ingredients of Seeds in Oil Tree Peonies

**DOI:** 10.3390/plants13091194

**Published:** 2024-04-25

**Authors:** Xihui Sun, Qingyu Zhang, Huiwen Zhang, Lixin Niu, Maifang Zhang, Yanlong Zhang

**Affiliations:** 1 College of Landscape Architecture and Arts, Northwest A&F University, Yangling 712100, China; sunxihui@nwafu.edu.cn (X.S.); zhangqingyu2020@163.com (Q.Z.); 15194032217@163.com (H.Z.); niulixin@nwsuaf.edu.cn (L.N.); 2 National Engineering Technology Research Center for Oil Peony, Northwest A&F University, Yangling 712100, China; 3 Shaanxi Academy of Forestry, Xi’an 710014, China

**Keywords:** oil tree peony, pollination methods, seed yield, oil content and quality, active ingredients of seeds

## Abstract

The tree peony, a novel woody oil crop extensively cultivated in China, necessitates further investigation into artificial pollination technology to enhance seed yield. In this study, we conducted artificial pollination experiments with 6-year-old *Paeonia ostii* ‘Feng Dan’ seedings for suitable pollen sources, pollen concentration, pollination timing, and pollination frequency. By evaluating seed yields, active ingredients, and oil quality, we derived the following significant conclusions. Firstly, compared to natural pollination, artificial pollination could significantly increase the fruit diameter by 13.94–27.58%, seed yields by 35.17–58.99%, and oil content by 6.45–7.52% in tree peonies. In active ingredients, seeds produced by pollen from Hantai County significantly enhanced starch content (by 48.64%), total phenols (by 41.18%) and antioxidant capacity (by 54.39%). In oil quality, seeds produced by pollen from Heyang County exhibited the highest α-linolenic acid and total fatty acid content with enhancements of 1.68%, 7.41%, and 8.48%. Secondly, hand pollination with pure pollen significantly increased seed yield by 58.99%, total phenol content by 40.97%, antioxidant capacity by 54.39%, and oil content by 1.53% compared to natural pollination. Thirdly, pollination at 2/3 bloom range significantly increased seed number by 63.08% and yield by 45.61% compared to natural pollination. Finally, the effect of one, two, and three pollination events had no difference in seed yield. So, to summarize, applying a 100% concentration of allochthonous pollen once is recommended when the bloom range is more than two thirds.

## 1. Introduction

**Figure 1:**
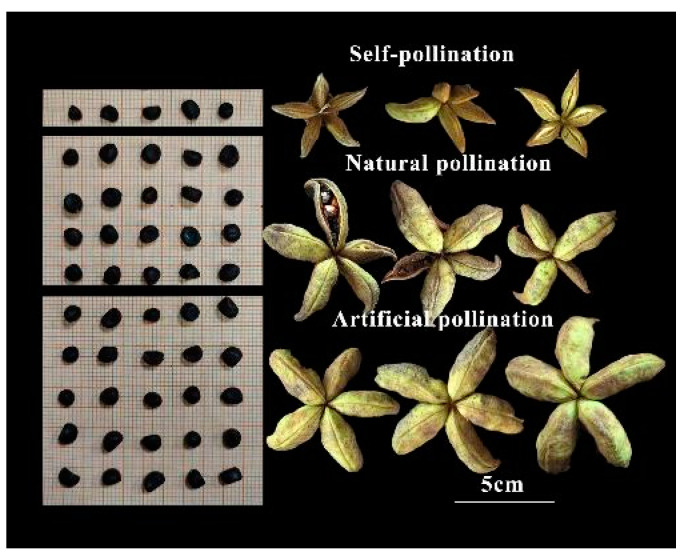
The seeds per fruit and fruit under different pollination methods in tree peonies (Natural pollination: pollination is carried out mainly by wind without human intervention; artificial pollination: cross-pollination through precise human intervention).

The tree peony (*Paeonia* section *Moutan* DC.) is a Chinese native plant cultivated for over 2000 years for its high ornamental, decorative, and medicinal value [1]. Recent studies have revealed that the seed oil of tree peonies is rich in unsaturated fatty acids, aligning with the current trend of healthy dietary habits. Consequently, large-scale cultivation and promotion of this plant have ensued [2,3]. *Paeonia ostii* and *Paeonia rockii* are currently the primary species of oil tree peonies being promoted in China. These species have been extensively planted across approximately 129,000 hectares in Shandong, Shaanxi, Henan, and Gansu Provinces to facilitate their widespread adoption. This endeavor yields an annual output of 530 k tons of seed oil. As a result of these efforts aimed at enhancing national food and oil security concerns within China’s agricultural landscape, the oil tree peony is gradually emerging as an indispensable woody oilseed crop. However, low seed yields remain the main obstacle impeding the development of the oil tree peony industry. As shown in Figure 1, the number of naturally pollinated seeds in the tree peony was significantly lower than those of artificial pollination.

Pollination plays a pivotal role in the production of fruits and seeds in agroecosystems [4]. Studies have indicated that over 70% of plants enhance their yield and efficiency through pollination [5], with global economic benefits from pollination ecosystem services exceeding USD 500 billion [6]. *P. ostii* ‘Feng Dan’ is a monoecious plant species featuring bisexual flowers. Stamens and petals serve as the reproductive organs critical to the fertilization process. Initially unreceptive, the pistil stigmas become receptive as the petals unfold and stamens release pollen, resembling hermaphroditism. The expression time of male and female sexual functions overlapped most, which provided the possibility of self-pollination. But, they exhibit significantly lower fruiting rates in self-pollination than after cross-pollination, while parthenogenesis does not occur naturally [7]. Therefore, emphasizing the importance of pollination has become crucial in addressing the issue of low seed yield in oil tree peonies.

It is widely acknowledged that pollen sources play a direct role in influencing the reproductive outcomes of the seeding plant [8]. This phenomenon has been observed across various crops [9], including hazelnuts [10], date palms [11], and almonds [12]. An experiment with 15 different kinds of pollen was conducted to demonstrate the direct effects on *P. ostii* ‘Feng Dan’ by Xie, who found that it exhibited significant differences in seed yields and oil content based on different pollen sources [13,14]. This result is of great significance for increasing tree peony production, but it cannot be applied to field production due to the scarcity of ornamental tree peony pollen. Insect pollination has been extensively studied as a critical method for enhancing crop yield in modern agriculture, with cherries [15], apples [16], and tomatos [17] being notable examples. Insect pollination has been found to significantly enhance both the rate and quality of fruit development, as evidenced by similar findings in *P. ostii* ‘Feng Dan’ cultivation [18]. Furthermore, investigations on the impact of different bee species on seed yield have revealed that honey bees exhibit superior pollination efficiency compared to bumble bees [19,20]. However, the tree peony lacks nectarines, and the cost of building sealed greenhouses and buying insects may far outweigh the benefits of increased yields, so it is necessary to explore new and feasible pollination methods. Despite numerous studies exploring tree peony pollination techniques, there is a lack of systematicity and practical applicability in farmland production. Therefore, it is imperative to summarize a standardized set of pollination techniques capable of significantly enhancing seed yield. Consequently, this study aims to conduct artificial pollination experiments using six-year-old *P. ostii* ‘Feng Dan’ seedings. By systematically examining various factors, such as pollen sources, pollen concentration, pollination timing, and frequency from multiple perspectives, this research intends to provide practical recommendations for the implementation of artificial pollination practices in the cultivation of oil tree peonies.

## 2. Materials and Methods

### 2.1. Test Site and Test Material

The trial was a field experiment in a tree peony garden located in Weinan City, Shaanxi Province, China (110°14′ E, 35°23′ N), from April to August 2022. The trial area had an average altitude of 721 m and experienced a temperate continental climate with an average annual temperature of 11.5 °C and rainfall of 553 mm. According to the Köppen climate classification, the trial area likely falls into a climate type such as Cwa, characterized by warm summers and cold winters, with moderate annual precipitation. The native reproductive mode of *P. ostii* ‘Feng Dan’, reliant solely on seed propagation, represents an obligatory reproductive strategy. The plants used in this experiment were seedlings. *P. ostii* ‘Feng Dan’ plants were planted on the plain using sandy loam soil with a moisture content ranging from 8% to 10%. The planting density consisted of six-year-old *P. ostii* ‘Feng Dan’ plants at a rate of 27,000 plants per hectare. The plant material utilized in this study was *P. ostii* ‘Feng Dan’, an oil tree peony that is a perennial deciduous shrub. The oil tree peonies in this garden undergo standardized agricultural intensive production with uniform management practices, ensuring that they flower in April and bear fruit in August. After six years of flowering age, the oil tree peony enters its high-yielding period. Before the experiment, the basic physicochemical properties of the soil (0–20 cm layer) were determined, according to Bao, and are described in the table below (Table 1) [21].

Three types of pollen are used: pollen of *P. ostii* ‘Feng Dan’ from Heyang County, pollen of *P. ostii* ‘Feng Dan’ from Hantai County, and mixed pollen from Yangling County. Among them, the mixed pollen was collected from 22 kinds of different cultivars. In this experiment, the purpose of picking mixed pollen for pollination was only to consider the profitable availability of pollen, so we did not count the specific proportion of each pollen in particular detail. The composition of the mixed pollen is about *P. ostii* ‘Feng Dan’ pollen/other ornamental pollens = 1:1. Pollen samples for experimentation were prepared by collecting anthers from various tree peony varieties during the flowering period. These anthers cracked in a climatic chamber set at 25 °C and 70% humidity. After the anthers split, the pollen is carefully collected in brown reagent bottles and stored in a 4 °C refrigerator. Additionally, a small amount of color-changing silica gel was added to each reagent bottle to prevent moisture absorption at low temperatures. Before use, the pollen was activated by allowing it to reach room temperature for 2 h, restoring its vitality. Collected pollen is stored at low temperatures for no more than two weeks. We checked that pollen viability was normal using the solid culture method before use [22]. A diluent primarily consisting of natural stone pine powder, which possesses a specific gravity compatible with pollen, was added, thereby ensuring no detrimental impact on pollen activity. Its key objectives encompass augmenting the quantity of dilutable pollen employed, facilitating post-pollination marking effects, and promoting efficient utilization of pollen sources.

### 2.2. Experimental Design

The artificial pollination experiment was conducted as four single-factor experiments to explore the effects of pollen sources (experiment 1), pollen concentration (experiment 2), pollination timing (experiment 3), and pollination frequency (experiment 4) on seed yield, active ingredients, oil content, and fatty acid composition of seed oil. The pollen used in experiments 2, 3, and 4 was from Hantai County, which is about 500 km away from the test site. To apply the same amount of pollen, a new cotton swab was used to dip into the test pollen before each flower was pollinated evenly.

In experiment 1 (Table 2), four treatments, namely, PS0, PS1, PS2, and PS3, were established to find the suitable pollen source. These treatments represented using different pollen sources: PS0 (natural pollination without intervention), PS1 (pollination with pollen from Yangling County, which is about 261 km away from the test site, and its altitude is 435–563 m), PS2 (pollination with pollen from Hantai County, which is about 500 km away from the test site, and its altitude is 478–2039 m), and PS3 (pollination with pollen from Heyang County, which is the test site, and its altitude is 721 m). *P. ostii* ‘Feng Dan’ is a kind of plant with significant individual differences; so, to minimize such differences, we chose to conduct four treatments on the same plant. A total of 3 flowers were pollinated on each plant for each treatment for a total of 15 plants and 180 flowers. Each flower is pollinated once on the second and third day after the designation.

In experiment 2 (Table 3), six treatments were compared, namely, PC0 (natural pollination without intervention), PC1 (pollination with pollen concentration of 2%), PC2 (pollination with pollen concentration of 5%), PC3 (pollination with pollen concentration of 20%), PC4 (pollination with pollen concentration of 50%), and PC5 (pollination with pollen concentration of 100%). The detailed proportions of pollen and pollen diluent at each concentration are shown in Table 3. In this experiment, six treatments were carried out simultaneously on the same plants, with each treatment consisting of three flowers. We processed 10 plants and 180 flowers in total. The pollens used in experiment 2 were all *P. ostii* ‘Feng Dan’ pollens from Hantai County, and different concentrations of pollens were obtained by mixing pollens with pollen fillers.

In experiment 3 (Table 4), four treatments were established: PT0 (natural pollination without intervention), PT1 (pollination is completed once when the bloom range/proportion of blossoms per tree is less than 1/3), PT2 (pollination is completed once when the bloom range is less than 2/3), and PT3 (pollination is completed once when the bloom range is less than 3/3). Herein, we defined the term ‘Bloom range’ as the ratio of opening flowers to the total number of flowers on each plant. Hand pollination was conducted only once at every pollination timing, as shown in Table 4. Each bloom range had seven plants assigned for treatment.

$$Bloom\;range=\frac{Number\;of\;flowers\;blooming}{Number\;of\;flowers}\times 100\%$$

In experiment 4 (Table 5), five treatments were established, namely, PF0, PF1, PF2, PF3, and PF4. Seven tree peony plants with similar growth status were selected for each treatment. None of the experimental plants were manually de-sexed or bagged.

### 2.3. Fruit Collection and Analysis

The artificial pollination test required the collection of fruits from each treatment plant, which were put into different gauze pockets and labeled for subsequent yield calculation and quality determination. After shelling the seeds, the seed kernels were pulverized using an FW-400 AD high-speed universal pulverizer (Xinbode Instrument Co., Ltd., Tianjin, China), the samples were extracted and prepared using methanol, the total phenol content of the samples was determined using the forintol–colorimetric method [23], the total flavonoid content was determined using the aluminum chloride–colorimetric method [24], and the samples were used to scavenge the DPPH radical capacity of the samples to determine their antioxidant activity [25]. The starch content was determined by the anthrone colorimetric method [26], and the soluble protein was determined by the Cauloblue method [27]. Finally, the seed oil was extracted using supercritical CO_2_ extraction equipment (SFE-2 model; Applied Separations, Allentown, PA, USA) to calculate the oil content. The fatty acids of seed oil were analyzed using the TRACE 1310 GC-ISQ (Thermo, Germany), as described previously, and the experiment was repeated three times [14,28].

### 2.4. Statistics and Analysis

The experimental data were analyzed and processed using Microsoft Excel, SPSS 26.0, and Origin 2021 software for statistical analysis; Microsoft Excel 2016 was mainly used to organize the data, SPSS 26.0 was used for data analysis, and Origin 2021 was used for graphing. A one-way ANOVA was performed by SPSS 26.0 (Version 26.0, Chicago, IL, USA), and the significance of differences and multiple comparisons analyses were handled by Duncan’s test at *p* < 0.05. The data in the results represent the mean ± standard deviation.

## 3. Results

### 3.1. Different Pollen Source Treatments

**Figure 2:**
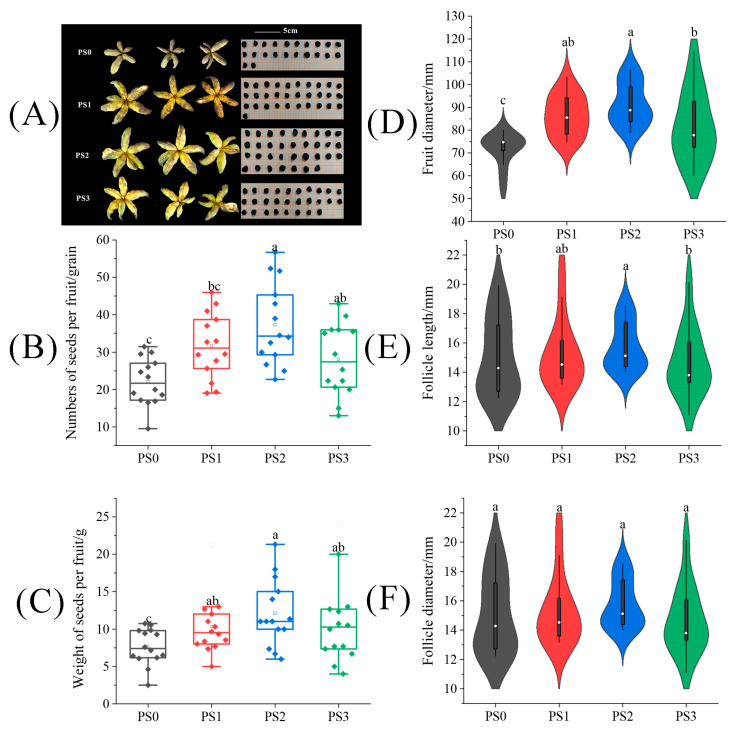
The difference of (**A**) fruits and seeds, (**B**) seed number per fruit, (**C**) seed weight per fruit, (**D**) fruit diameter, (**E**) follicle length, and (**F**) follicle diameter in tree peonies under different pollen source treatments. (PS0: natural pollination, PS1: pollinated by mixed pollen of 22 varieties from Yangling County, PS2: pollinated by pollen of *P. ostii* ‘Feng Dan’ from Hantai County, PS3: pollinated by pollen of *P. ostii* ‘Feng Dan’ from Heyang County). Different lowercase letters represent significant differences at the *p* < 0.05 level, and the same is below.

The fruit type of tree peonies is the follicle, with at least one or more seeds in each fruit. The fruit set rate always is 100% and is not suitable for our description of experimental results. So, we chose the number of fruits per fruit to show the effect of pollination. As shown in Figure 2A, although the situation of the fruit set in the tree peony pollinated by different pollen sources varied, all showed consistent improvement in fruit size and seed number compared to natural pollination treatment (PS0). In Figure 2D–F, fruit diameter and follicle length under the PS2 treatment showed significant enhancements of 27.57% and 16.51% over the PS0 treatment. However, there was no difference in follicle diameter among all treatments. Then, compared to the PS0 treatment, it can be seen in Figure 2B that the PS2 and PS3 treatments significantly increased the seed number per fruit by 68.78% and 44.91%, respectively. As shown in Figure 2C, the PS1, PS2, and PS3 treatments significantly increased seed weight by 35.17%, 59.06%, and 41.99%, respectively, compared with the PS0 treatment (*p* < 0.05).

**Figure 3:**
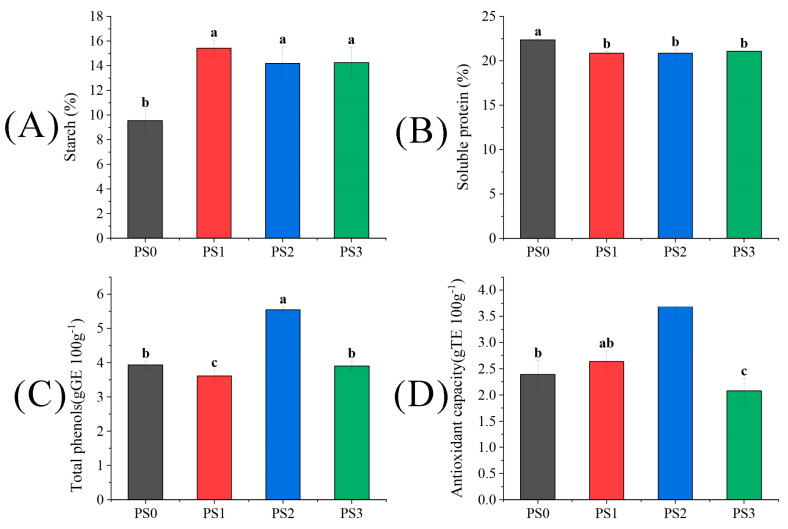
The difference of (**A**) starch, (**B**) soluble protein, (**C**) total phenols, (**D**) antioxidant capacity in tree peonies under different pollen source treatments (Different lowercase letters represent significant differences at the *p* < 0.05 level).

As depicted in Figure 3, the starch content of seeds under three hand pollination treatments was significantly higher than the PS0 treatment by 61.74%, 48.64%, and 49.37%, respectively, indicating a significant increase due to artificial pollination. Conversely, the soluble protein content exhibited an opposite trend to the starch content, with PS0 showing the highest effect. For the total phenols and antioxidant activity in the highest PS2 treatment, the enhancements over the PS0 treatment were 40.97% and 54.39%, respectively (*p* < 0.05).

We measured the oil content and the content of the five principal fatty acids: seed palmitic acid, stearic acid, oleic acid, linoleic acid, and alpha-linolenic acid under different pollen source treatments in Table 6. Oil contents under three artificial hand pollination treatments were all significantly elevated compared with the PS0 treatment. Among five fatty acids, only oleic acid content was significantly higher in the hand pollination treatments. Under the four treatments, the PS3 treatment had the highest content of stearic acid, oleic acid, linoleic acid, α-linolenic acid, and total fatty acids in seed oil, with enhancements of 13.99%, 14.23%, 10.72%, 7.41%, and 8.48% over the PS0 treatment (*p* < 0.05).

### 3.2. Different Pollen Concentration Treatments

**Figure 4:**
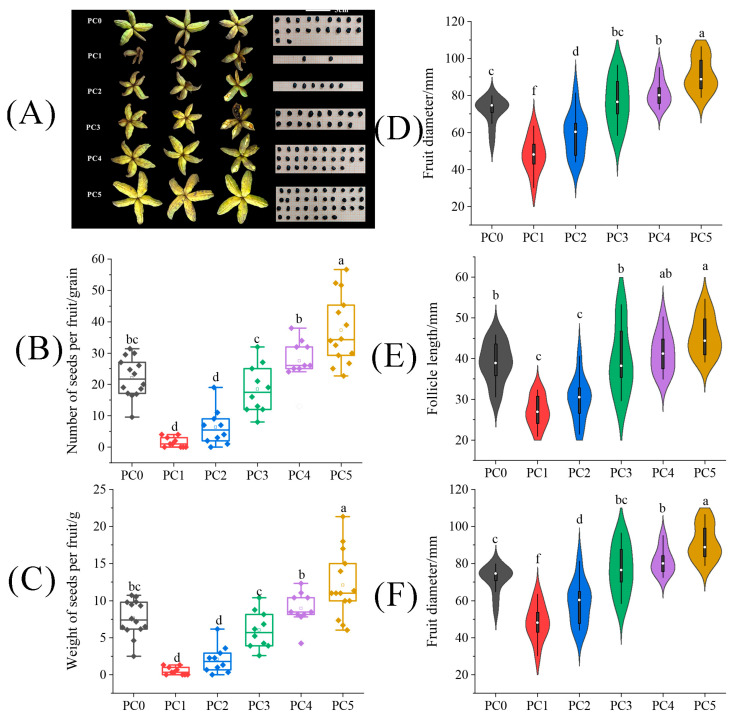
The difference of (**A**) fruits and seeds, (**B**) seed number per fruit, (**C**) seed weight per fruit, (**D**) fruit diameter, (**E**) follicle length, and (**F**) follicle diameter on tree peonies under different pollen concentrations. (PC0: natural pollination, PS1, pollinated by 2% pollen, PS2: pollinated by 5% pollen, PS3: pollinated by 20% pollen, PS4 pollinated by 50% pollen, PS5: pollinated by 100% pollen). Different lowercase letters represent significant differences at the *p* < 0.05 level.

The shape and size of the fruit and seed number treated with pollen of different concentrations are shown in Figure 4A. In Figure 4C, pollen concentration is positively correlated with seed number and yield. Compared with the PC0 treatment, the number and weight of seeds per fruit under the PC5 treatment were significantly increased by 68.76% and 58.99%. Through Pearson correlation analysis, it was found that pollen concentration was significantly positively correlated with fruit diameter (r = 0.76), follicle length (r = 0.70), and follicle diameter (r = 0.71) (Figure 4D–F). PC5 treatment represents the treatment with the highest pollen concentration, so the fruit diameter and follicle length under this treatment were significantly higher than the PS0 treatment, with an increased amplitude of 27.58% and 16.53%, respectively (*p* < 0.05).

**Figure 5:**
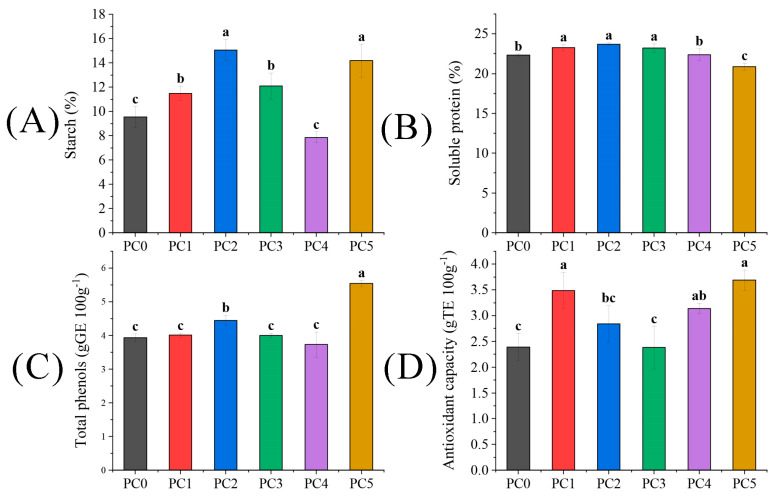
The difference of (**A**) starch, (**B**) soluble protein, (**C**) total phenols, and (**D**) antioxidant capacity in tree peonies under different pollen concentration treatments. Different lowercase letters represent significant differences at the *p* < 0.05 level.

The content of active ingredients of seeds was measured in absolute values (Figure 5). The trends of starch and soluble protein in kernels varied with the increase in pollen concentration (Figure 5A,B). Initially, the starch content exhibited an increasing trend followed by a decreasing trend before ultimately showing growth. The highest points were observed in the PC2 and PC5 treatments, with ranges of 57.76% and 48.64% higher than the PC0 treatment. Conversely, the soluble protein initially increased continuously, followed by a decrease. Among these treatments, PC5 had the highest starch content while exhibiting the lowest soluble protein content. In Figure 5C, the PC1 and PC5 treatments significantly improved total phenol content by 13.23% and 40.97%, respectively, compared to the PC0 treatment (*p* < 0.05). The antioxidant capacity under the PC1, PC4, and PC5 treatments was significantly higher than PC0, exhibiting increases of 46.03%, 31.38%, and 54.39%, respectively (*p* < 0.05).

The seed oil content was significantly improved under the PC3 and PC5 treatments, with a significant increase of 5.51% and 6.85%, respectively, compared to the PC0 treatment (Table 7). In terms of the fatty acid composition of the seed oil, α-linolenic acid exhibited the highest content in the PC1 treatment, while the other four fatty acids were most abundant in the PC4 treatment. The total content of fatty acids displayed a decreasing then increasing trend with increasing pollen concentration and reached its maximum at the PC5 treatment. Although there was a slight elevation compared to the PC0 treatment, it was not statistically significant. Furthermore, the seed oil content demonstrated an upward trend as pollen concentration increased, reaching its peak at the PC5 treatment, surpassing that of PC0 by 6.85% (*p* < 0.05).

### 3.3. Different Pollination Timing Treatments

**Figure 6:**
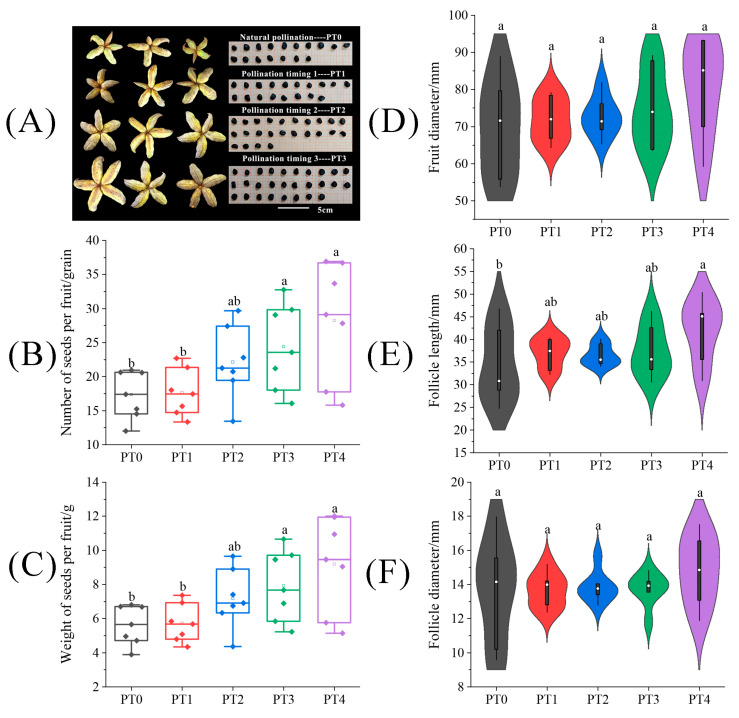
The difference of (**A**) fruits and seeds, (**B**) seed number per fruit, (**C**) seed weight per fruit, (**D**) fruit diameter, (**E**) follicle length, and (**F**) follicle diameter on tree peonies under different pollination timing treatments. Different lowercase letters represent significant differences at the *p* < 0.05 level.

Under different pollination timing treatments, the fruit size traits, seed number, and weight of tree peonies are shown in Figure 6. The fruits and seeds harvested under the different treatments were photographed, counted, and weighed (Figure 6A–C). The results presented in Figure 6 demonstrated a positive correlation between pollination timing and seed yields. The differences in fruit appearance traits among different pollination timing treatments were mainly observed in follicle length. In Figure 6E, we can learn that larger follicle length was associated with delayed pollination timing, and the follicle length under the PT3 treatment was significantly enhanced by 21.46% compared to the PT0 treatment. For the number and weight of seeds per fruit shown in Figure 6D,E, these parameters were significantly enhanced by 63.08% and 45.61%, respectively, under the PT3 treatment compared to the PT0 treatment, (*p* < 0.05).

### 3.4. Different Pollination Frequency Treatments

**Figure 7:**
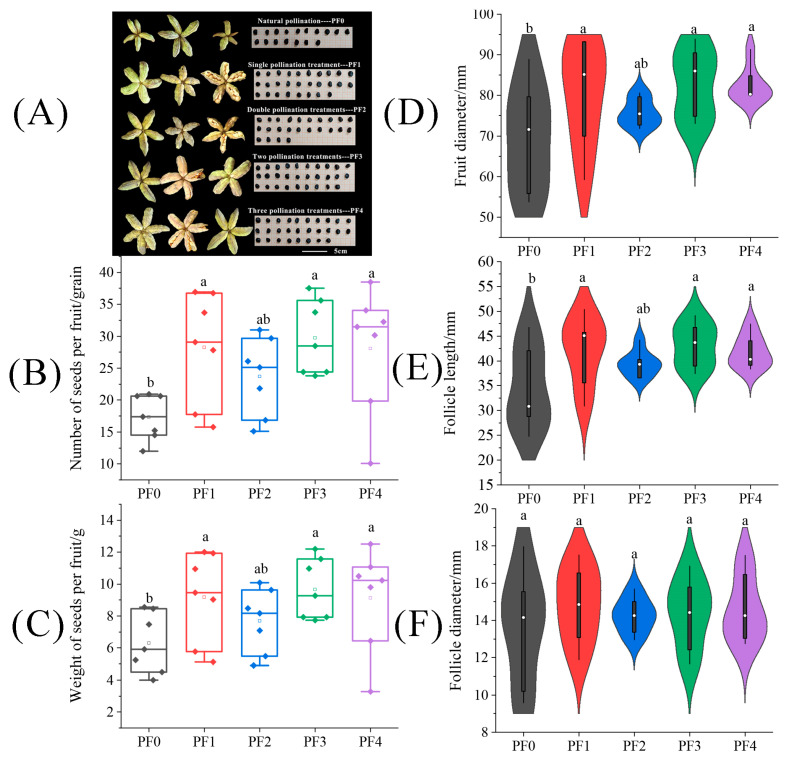
The difference of (**A**) fruits and seeds, (**B**) seed number per fruit, (**C**) seed weight per fruit, (**D**) fruit diameter, (**E**) follicle length, and (**F**) follicle diameter on tree peonies under different pollination frequency treatments. (Different lowercase letters represent significant differences at the *p* < 0.05 level).

The results presented in Figure 7 indicated that the fruit size across different pollination frequency treatments primarily focused on fruit diameter and follicle length. In Figure 7D,E, it is evident that including the number of seeds per fruit, the weight of seeds per fruit, fruit diameter, and follicle length under three treatments, (PF1, PF3, and PF4) were significantly higher than the PF0 treatment. There was no significant difference in follicle diameter between treatments. Notably, although the tree peony under the PF1 treatment underwent only one pollination, it significantly enhanced the weight of seeds per fruit by 45.61% compared to the PF0 treatment. Meanwhile, the number of seeds per fruit, fruit diameter, and follicle length under the PF1 treatment significantly increased by 61.86%, 18.75%, and 20.91%, respectively (*p* < 0.05).

## 4. Discussion

### 4.1. Effects of Different Pollen Sources on Tree Peonies

The breeding system plays a pivotal role in determining oil content and seed yield. Tree peonies are characterized as monoecious and heterogamous plants, exhibiting a low fruiting rate through self-pollination or sympatric pollination mechanisms. They primarily rely on insect-mediated pollination by bees for successful fertilization [29,30]. Since no nectar secretion was observed in the floral organs of *P. ostii* ‘Feng Dan’, pollen serves as the sole incentive for attracting insect pollinators to visit flowers in search of valuable nutrients. Therefore, with practical implications in mind, we procured three readily available types of pollen and conducted a comprehensive investigation into their impact on the seed yield of *P. ostii* ‘Feng Dan’, aiming to provide valuable insights for the potential commercialization of seed oil production.

Although *P. ostii* ‘Feng Dan’ exhibits a high natural fruit set rate, the assessment of its fruitfulness is often based on the number and size of seeds in the follicles. In this study, we observed that under three artificial hand pollination treatments, *P. ostii* ‘Feng Dan’ displayed significantly improved seed yield, active ingredients of seeds, and oil quality compared to natural pollination. These findings align with previous research by Si [31], Han [7], and other scholars. The observed phenomenon coincided with the documented decline in hickory and hazelnut inbreeding [32], wherein self-pollinated kernels of hickory and hazelnut exhibited reduced weight and size compared to those resulting from cross-pollination [33,34]. The PS3 treatment had no significant difference in improving seed weight compared with PS1 and PS2, which indicated that mixed pollen was not needed in field production. This may be related to the uneven pollen activity of various varieties of tree peonies (4.78–78.62%). Therefore, when preparing pollen for field peony production, attention should be paid to the selection of varieties with high pollen activity.

Furthermore, the oil content and soluble protein of *P. ostii* ‘Feng Dan’ seeds were influenced to varying degrees by different pollen sources. Specifically, tree peonies subjected to artificial pollination exhibited higher oil content than those in their natural state; however, the soluble protein content was significantly lower than that of the control group. A distinct negative correlation between oil yield and soluble protein was observed. This phenomenon has also been reported in other oilseed crops, such as peanut [35], oil tea [36], and rape [37]. The speculation arises from the competition for a common synthetic substrate between protein and oil compounds. Various pollen sources provide additional nutrients that influence the rate of protein accumulation, subsequently affecting the overall protein content in seeds [38].

The composition of the five primary fatty acids in the seed oil was influenced by different pollen sources, underscoring the importance of selecting appropriate pollen sources to enhance the fatty acid profile of tree peony seed oil. Similar findings were reported for almonds [39]. Previous studies have also demonstrated that varying pollen sources can impact the chemical composition of fruits, such as altering soluble solid content in date palm fruits and bitter amygdalin levels in almonds [11,39]. Additionally, it should be noted that artificial emasculation of flowers was carried out before hand pollination treatments, and the stamens have been proven to have an important impact on the fruit setting of *P. ostii* ‘Feng Dan’ in previous studies [40]. Since large-scale emasculation does not occur in field plants, we are more confident in the pollen of allochthonous plants screened, which is conservatively estimated to increase seed yield by 58.99%.

### 4.2. Effects of Different Pollen Concentrations in Tree Peonies

The present study revealed a noteworthy improvement in seed characteristics, such as fruit size and the number and weight of seeds per fruit of *P. ostii* ‘Feng Dan’, with increasing pollen concentration. This suggests that the higher pollen concentration led to an increased number of fertilized ovules, aligning with Sanchez’s findings in his oil tea research [41]. In most heterogamous pollination-dependent plants, the pistil must receive adequate pollen to ensure a successful seed set [42,43]. This explanation is further supported by the significantly lower seed yields observed under low pollen concentration treatments compared to naturally pollinated conditions in this experiment. The number and weight of seed grains gradually increased in response to the elevated pollination concentration, aligning with the established conclusion that cocoa seed count is directly proportional to fertilization within specific ranges [44].

The fruit size in tree peonies initially increased with rising pollination concentration but subsequently declined. This trend aligns with previous findings in *Ginkgo biloba*, where reduced pollination concentration resulted in enhanced longitudinal diameter, transverse diameter, and thickness of fruit seed kernels [45]. Lower pollination concentration leads to fewer fertilized ovules, as it allows nutrient allocation towards a limited number of ovules within the tree peony system, thereby promoting greater total seed plumpness. Notably, for improving seed quality enhancement purposes, maintaining a specific range characterized by low pollen concentration has been identified as beneficial for optimizing the quality attributes associated with tree peony seeds.

### 4.3. Effects of Different Pollination Timing in Tree Peonies

This study identified an optimal pollination period for achieving higher seed yields of *P. ostii* ‘Feng Dan’, occurring when two thirds of the flowers are opening. This finding aligns with previous research on pepper hybridization, indicating that the middle and late stages resulted in higher seed yield and production efficiency, as observed in chili peppers and other crops, such as broccoli [46]. However, it is crucial to note that the optimal pollination period may vary among different plant species. For instance, self-incompatible broccoli achieves higher pod yield, number of seeds per fruit, thousand-grain seed quality, and overall seed yield when pollinated towards the end of flowering for early maturing varieties or during the entire bloom stage for late-maturing ones. Additionally, improper storage conditions that can reduce the viability of harvested pollen may be reduced due to improper storage conditions, which, in turn, significantly affect plant fruit yield, a phenomenon also relevant to oil tree peonies [47]. Lower seed yields translate to decreased oil production in *P. ostii* ‘Feng Dan’. In this investigation, delayed pollination timing is often correlated with increased follicle length. However, this association did not imply a positive influence of later flowering or higher temperatures on seed production. Instead, a delayed pollination period, attributed to a broader flowering amplitude, significantly enhances the probability of pollen deposition on the stigma. Consequently, this leads to a greater number of fertilized ovules within the peony fruit, thereby augmenting follicle length. Furthermore, it is essential to consider additional factors, such as climatic conditions and the risks associated with delayed pollination caused by pests and diseases.

### 4.4. Effects of Different Pollination Frequency in Tree Peonies

The duration of stigma receptivity varies among different plant species, ranging from more than ten days to only a few hours, a trait often associated with the flowering period [48]. Through our observations, we noted that the secretion of mucus indicated the peak stigma receptivity in tree peony flowers, occurring from the initial to full bloom stages at the individual flower level. Tree peonies exhibit uneven flowering patterns within a single plant, making multiple pollinations advantageous in increasing the likelihood of pollen-stigma encounters and subsequent successful pollination. Previous studies have shown that repetitive pollination enhanced fruit yield across diverse crop species, such as tomatos [49], eggplants [50], and black-skinned winter melons [51]. However, our study did not find significant differences in seed yield and the number of *P. ostii* ‘Feng Dan’ seeds among different pollination frequency treatments. Some researchers concluded that repeated pollination using a 1/5 concentration of pollen greatly increased the number and seed yield of apples [52]. So, we speculate that the lack of significant improvement in seed yield in our study may be attributed to the specific role of pure pollen in facilitating the pollination process for *P. ostii* ‘Feng Dan’, where one round of pollination may suffice to fertilize most flowers successfully. Conducting multiple pollinations during this period might not significantly increase the seed yield. In comparison with the repeated pollination of the ‘Fuji’ apple, our study found that pollination with a high concentration of pollen during the appropriate flowering period achieved the same results, which is more time saving and labor saving.

## 5. Conclusions

This study proposed a series of pollination techniques focusing on pollen sources, pollen concentration, pollination timing, and pollination frequencies in oil tree peony production in the field. Through field tests, we established that heterologous pure pollen was the most suitable, and optimal pollination timing occurred when the flowering range exceeded 2/3. Furthermore, we elucidated the practical importance of repeated pollination in enhancing seed yield in tree peony cultivation.

## Figures and Tables

**Table 1:** Physicochemical properties of the soil before the experiment.

Physicochemical Property	pH	SOM (g kg^−1^)	TN (g kg^−1^)	AN (mg kg^−1^)	AP (mg kg^−1^)	AK (mg kg^−1^)
CK	8.05	14.76	0.50	10.82	22.00	60.00

**Table 2:** The introduction of pollen sources and the number of pollination events in practice 1.

Treatments	Pollen Source	Number of Pollination Events
PS0	Natural pollination	0
PS1	Mixed pollen of 22 varieties from Yangling County	2
PS2	*P. ostii* ‘Feng Dan’ from Hantai County	2
PS3	*P ostii* ‘Feng Dan’ from Heyang County	2

**Table 3:** The introduction of pollen concentration and the number of pollination events in experiment 2.

Treatments	Pollen Concentration	Number of Pollination Events	Ratio of Pollen to Filler
PC0	Natural pollination	0	/
PC1	2%	2	1:49
PC2	5%	2	1:19
PC3	20%	2	1:4
PC4	50%	2	1:1
PC5	100%	2	/

**Table 4:** The introduction of pollination timing and the number of pollination events in experiment 3.

Treatments	Pollination Timing	Number of Pollination Events
PT0	Natural pollination	0
PT1	Bloom range ≤ 1/3	1
PT2	1/3 < bloom range ≤ 2/3	1
PT3	2/3 < bloom range	1

**Table 5:** The introduction of pollination frequency and the number of pollination events in experiment 4.

Treatments	Pollination Timing	Number of Pollination Events
PF0	Natural pollination	0
PF1	(2/3, 1]	1
PF2	(0, 1/3] and (1/3, 2/3]	2
PF3	(1/3, 2/3] and (2/3, 1]	2
PF4	(0, 1/3], (1/3, 2/3], and (2/3, 1]	3

Note: PF1 treatment refers to pollination when the flowering range is (2/3, 1], PF2 treatment refers to one pollination each at flowering ranges of (0, 1/3] and (1/3, 2/3], PF3 treatment refers to one pollination each at flowering ranges of (1/3, 2/3] and (2/3, 1], PF4 treatment is pollinated once each at (0, 1/3], (1/3, 2/3], and (2/3, 1] flowering stages.

**Table 6:** Effects of different pollen sources on oil content and fatty acid composition of seed oil (The comparisons are made for each file; different lowercase letters represent significant differences at the *p* < 0.05 level, and the same is below).

Treatments	PS0	PS1	PS2	PS3
Oil content (%)	22.33 ± 0.64b	23.77 ± 0.79a	23.86 ± 0.23a	24.01 ± 0.66a
^a^ Palmitic acid	4.65 ± 0.03b	4.47 ± 0.02c	4.81 ± 0.03a	4.60 ± 0.00b
Stearic acid	1.43 ± 0.01c	1.49 ± 0.01b	1.44 ± 0.01c	1.63 ± 0.00a
Oleic acid	12.37 ± 0.08c	11.80 ± 0.07d	12.75 ± 0.08b	14.13 ± 0.00a
Linoleic acid	13.15 ± 0.09d	13.91 ± 0.09c	14.27 ± 0.09b	14.56 ± 0.00a
α-Linolenic acid	30.68 ± 0.20b	29.92 ± 0.18c	30.40 ± 0.20b	32.63 ± 0.00a
Total fatty acid	62.28 ± 0.41c	61.58 ± 0.37c	63.66 ± 0.40b	67.56 ± 0.00a

^a^ Content of major fatty acids in seed oil (g/100 g crude oil), and the same is below.

**Table 7:** Effects of different pollen concentrations on oil content and fatty acid composition of seed oil. (Different lowercase letters represent significant differences at the *p* < 0.05 level.)

Treatments	PC0	PC1	PC2	PC3	PC4	PC5
Oil content (%)	22.33 ± 0.64bc	21.34 ± 0.02c	21.35 ± 0.62c	23.56 ± 0.13a	22.82 ± 0.23ab	23.86 ± 0.23a
Palmitic acid	4.65 ± 0.03c	4.34 ± 0.05d	4.18 ± 0.07e	4.45 ± 0.13d	5.05 ± 0.05a	4.81 ± 0.03b
Stearic acid	1.43 ± 0.01b	1.45 ± 0.02b	1.07 ± 0.02c	1.37 ± 0.04c	1.49 ± 0.02a	1.44 ± 0.01b
Oleic acid	12.37 ± 0.08c	12.24 ± 0.14c	10.38 ± 0.18e	11.76 ± 0.36d	13.19 ± 0.14a	12.75 ± 0.08b
Linoleic acid	13.15 ± 0.09b	11.24 ± 0.13c	9.95 ± 0.17d	13.18 ± 0.40b	14.57 ± 0.16a	14.27 ± 0.09a
α-Linolenic acid	30.68 ± 0.20b	32.6 ± 0.38a	27.47 ± 0.45d	27.44 ± 0.83d	29.33 ± 0.32c	30.4 ± 0.20b
Total fatty acid	62.28 ± 0.41ab	61.86 ± 0.73b	53.05 ± 0.88d	58.2 ± 1.77c	63.63 ± 0.69a	63.66 ± 0.40a

## Data Availability

The data is contained within the manuscript.
